# Should aromatase inhibitors be used as initial adjuvant treatment or sequenced after tamoxifen?

**DOI:** 10.1038/sj.bjc.6602964

**Published:** 2006-01-24

**Authors:** J Cuzick, P Sasieni, A Howell

**Affiliations:** 1 Cancer Research UK Centre for Epidemiology, Mathematics and Statistics, Wolfson Institute of Preventive Medicine, Barts and the London, Queen Mary's School of Medicine and Dentistry, Charterhouse Square, London EC1M 6BQ, UK; 2 Cancer Research UK Department of Medical Oncology, Christie Hospital NHS Trust, Wilmslow Road, Manchester M20 4BX, UK

**Keywords:** aromatase inhibitors, breast cancer, adjuvant treatment, sequencing schedules

## Abstract

A number of trials have studied the value of aromatase inhibitors (AIs) for the adjuvant treatment of early hormone-responsive postmenopausal breast cancer. Three different AIs have been used and they have been compared as initial treatment (two trials) or after 2–3 years of tamoxifen (four trials), in both cases against a standard arm of 5 years of tamoxifen. In addition, two trials have evaluated AIs against no treatment after 5 years of tamoxifen. In all circumstances, the AIs have demonstrated superior efficacy. However, no results are currently available for the key question, that is – is it better to start initially with an AI or use it sequentially after 2 years of tamoxifen? Here, we review the trial results and present two models, which address this issue. The models clearly show that early treatment with an AI is superior to using it after 5 years of tamoxifen. They also favour an upfront strategy to sequencing after 2 years of tamoxifen, but in this case the differences are small and model-dependent. A key question is whether AIs have substantially better efficacy than tamoxifen for ER-positive–PgR-negative tumours, where the data are currently contradictory. A mechanism explaining why greater efficacy might be so is proposed. Further results from ongoing trials will be needed to resolve this issue.

## BACKGROUND

Several trials have shown that the aromatase inhibitors (AIs) achieve a lower recurrence rate than tamoxifen when used as adjuvant treatment for post-menopausal women with hormone-receptor-positive breast cancer. Interpretation of these results is complicated by the fact that three different AIs have been used, and also that they have been offered at different times in the treatment programme. Three types of schedule can be identified: initial treatment; sequencing treatment after 2–3 years of tamoxifen; and extended treatment after 5 years of tamoxifen. Larger reductions in the hazard ratio for recurrence have been reported in the sequencing and extended treatment regimens, but a proper evaluation of the overall impact on recurrence needs to account for the higher recurrence rates that occurred in the period before switching to an AI.

Here, we briefly review the design and findings of these trials and use the results to develop models, which can be used to explore the long-term (10 year) impact of different approaches. The two types of model used are: (i) a surface model, which uses the available data in the most straightforward way to predict outcomes; and a so-called ‘deep’ model which tries to explain the current data by an underlying mechanistic model. The goal of this later model is to try to explain and understand at a biologic level what has been observed. If correct, it may provide more accurate long-term predictions of recurrence rates. The model also identifies some key questions that should be amenable to study in the current trials.

## METHODS

### End points

Several end points could be used in evaluating the different strategies. Recurrence is the most sensitive endpoint for evaluating efficacy, and includes distant recurrence, local recurrence and contralateral tumours. This is preferable to disease-free survival (DFS), which would also include intercurrent deaths without recurrence. These latter events rightfully belong in the safety or overall analysis, but are not relevant for efficacy, where they dilute real differences in efficacy with events unrelated to breast cancer. Using only distant recurrences would provide a better surrogate for death from breast cancer, but their use requires longer follow-up, and it is too early to use this end point at this stage.

The use of recurrence rates gives equal weight to early and late recurrences. It is clear that an early recurrence is more detrimental than a late one, and integrating the recurrence curves to give the per cent of ‘time lost to recurrence’ is probably the best summary measure of efficacy in this context. Thus a recurrence at year 1 would lose 90% of the first 10 years to recurrence, but a recurrence at year 9 would only lose 10%. For a 10-year window, the actual number of years lost is just the percentage multiplied by 10, and this gives a useful measure for comparing strategies. We will evaluate both measures, but focus on this latter outcome measure below.

### Carryover

One key question is how long the effect will be maintained after treatment is stopped. The EBCTCG overview has shown that the effect of tamoxifen on recurrence is maintained for about 5 years after stopping 5 years of treatment (Early Breast Cancer Trialists' Collaborative Group, 2005). The only data on AIs come from the ATAC trial where the same magnitude of reduction was seen in year 6 as in previous years (ATAC Trialists' Group, 2005).

Carryover is a consequence of curing a proportion of patients, and not simply holding tumours in check. If treatment is curative, then all future recurrences are prevented, so the hazard ratio will remain below unity even after treatment has stopped. This is expected for chemotherapy, but the long-term results from the overview strongly suggest that tamoxifen is also curing a proportion of patients, and that its action is more than cytostatic. A reasonable assumption is that the early superiority of AIs over tamoxifen will result in a higher cure rate and that the reduction in recurrence rates will also be maintained for 5 years after the treatment ceases. This assumption is used in our base case model. However, given the importance of this parameter and the paucity of data, models with a 2-year carryover are also evaluated.

### Available trial data

The reported adjuvant trials have used three different AIs in three different settings. The key parameters for the different trials are summarized in Table 1. Trials are grouped into initial treatment trials, sequencing or switching trials, and extended treatment trials. Where available, hazard ratios are given (or estimated) separately according to progesterone receptor (PgR) status. We first estimated baseline recurrence rates for oestrogen receptor (ER)-positive patients treated with 5 years of tamoxifen. The hazard rates for recurrence are known to peak in the second year of follow-up and then decline. We have used the results of the ATAC to model these rates in the first 6 years, giving annual rates of 1.5, 3.8, 2.6, 2.9, 2.5, and 3.1 per cent for years 1–6, respectively. The overview data have then been used for years 7–10 and a constant rate of 2.4% year^−1^ provides an excellent fit to these data (Early Breast Cancer Trialists' Collaborative Group, 2005).

### Initial treatment trials

The hazard ratio for anastrozole in the ATAC trial was 0.74. (ATAC Trialists' Group, 2005) and for letrozole in the BIG1-98 trial was 0.72 (BIG1–98 Collaborative Group, 2005), suggesting a similar overall effect. We have used an HR of 0.73 in our analysis.

### Sequencing trials

For studies of the use of an AI after 2–3 years of tamoxifen, hazard rates of 0.70 have been reported for the IES study using exemestane (Coombes *et al*, 2004a, 2004b), 0.60 for the ABCSG8/ARNO95 trial (Jakesz *et al*, 2005a) and 0.46 for the latest update of the much smaller Italian trial using anastrozole in node-positive women only (Boccardo *et al*, 2005). In this model, we have given equal weight to the two large trials and assumed a hazard ratio of 0.65 beginning after 2 years of tamoxifen.

### Extended treatment trials

The MA-17 trial was the first trial to report on the use of an AI (letrozole) after 5 years of tamoxifen (Goss *et al*, 2003, 2005). Although intended for a 5-year period of use, the trial was stopped early with a 29-month median follow-up and a hazard ratio of 0.57. Recently an Austrian study (ABCSG Trial 6a) has also reported on the randomization to 3 years of anastrozole *vs* placebo after 5 years of tamoxifen (with or without aminoglutethemide). They found a hazard ratio of 0.64 for extended treatment (Jakesz *et al*, 2005b). We have taken an average effect of 0.60, and assume this effect will persist for the full treatment period plus a carryover period as above.

For these trials, the treatment period extends beyond 5 years, so a fairer comparison would be against initial AI or sequencing strategies of the same duration. However, by assuming a 5-year carryover effect, this will have little impact on the results for the first 10 years.

## RESULTS

### Surface model

**Figure 1:**
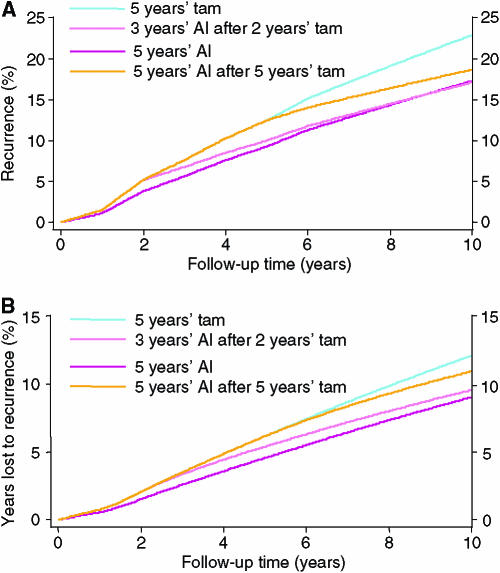
Proportion of oestrogen-receptor-positive women who will develop recurrence (**A**) and time lost to recurrence (**B**) in the first 10 years of follow-up for four different treatment strategies using the ‘surface model’ and a 5 year carryover effect: aromatase inhibitor for 5 years; 2 years of tamoxifen followed by 3 years of an AI; 5 years of tamoxifen followed by 5 years of an AI; and 5 years of tamoxifen alone. See text for parameter estimates.

The results predicted from this model are shown in Figure 1 and Table 2 for the first 10 years of follow-up. Table 2 also gives results for a 2-year carryover period. For a 5-year carryover period, the absolute reduction in recurrence rate at 10 years is 5.6% for initial use of an AI compared to 5 years of tamoxifen (22.9 *vs* 17.3%), leading to 1 less recurrence for every 18 women treated. The average time lost to recurrence is reduced by 3.1% or 3.7 months. The model predicts a similar recurrence rate at 10 years for the strategy of switching to an AI after 2 years compared as to initial use (Figure 1A); but, because the benefits occur later, there are still more years of life lost to recurrence with this strategy (Figure 1B, 9.6 *vs* 9.0%, corresponding to a 0.7 month difference). Waiting 5 years before commencing an AI is not an effective strategy; although for women who have already received 5 years of tamoxifen, it is more effective than stopping treatment.

### PgR subgroups

Some but not all trials have suggested that the benefit of AIs over tamoxifen is particularly apparent in the ER-positive but PgR-negative subgroup. This was particularly apparent in the ATAC trial where the hazard ratio was 0.43 for PgR− patients (*n*=880) and 0.83 for PgR+ patients (*n*=3834) (Dowsett *et al*, 2005). Smaller differences in this direction were seen in the ARNO/ABCSG trial again using anastrozole, where the effect on PgR− tumours (*n*=564) was again very large (HR=0.42) (Jakesz *et al*, 2005a), and to a lesser extent in the IES trial using exemestane (Coombes *et al*, 2004a). However, no differences were seen in the early report of the BIG 1–98 trial using letrozole (Table 1). Data on PgR subgroups are not available for the other trials.

If one uses a hazard ratio of 0.43 for anastrozole for ER+/PgR− patients, as reported in both the ATAC and ABCSG8/ARNO95 studies (ATAC Trialists' Group, 2005; Jakesz *et al*, 2005a), the indication for using an AI initially in the subgroup is very strong, with very large gains both in 10 year recurrence rates (16.3 *vs* 20.2 *vs* 34.0%) and total time lost to recurrence (8.5 *vs* 12.3 *vs* 18.4%), leading to gains of 4.6 and 11.9 months, respectively, when compared with sequencing after 2 years of tamoxifen or use of tamoxifen only.

Concomitant with a greater effect of anastrozole in the ER+/PgR− group in the ATAC trial is a less extreme hazard ratio in the ER+/PgR+ group of 0.84 (Dowsett *et al*, 2005). If this is a true estimate of the difference in this subgroup and the hazard ratio for an AI starting after 2 years of tamoxifen is 0.65 and after 5 years is 0.60, then a switching strategy after 2 years dominates an AI upfront strategy for this subgroup, both in terms of recurrence rates at 10 years (15.2 *vs* 13.2%) and time lost to recurrence (0.6 months difference; Table 2). Both of these regimens still dominate a strategy of waiting 5 years to commence use of an AI in terms of time lost to recurrence (Table 2).

However, purely statistical ‘surface models’ can be unreliable for long-term extrapolation, and a more biologic ‘deep model’ suggests a somewhat different picture, especially for the ER+/PgR+ subgroup.

### Deep model

**Figure 2:**
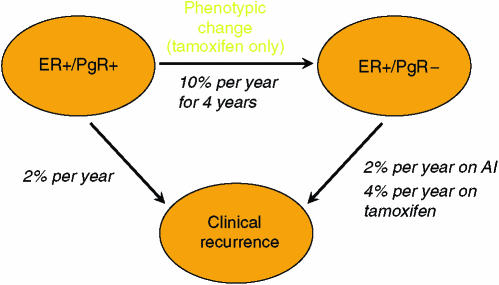
Schematic representation of the ‘deep model’. Patients with ER+/PgR+ develop recurrence at a rate of 2% year^−1^ while the micrometastases retain this phenotype, regardless of whether treated by tamoxifen or an AI, but tamoxifen-treated patients transition to the ER+/PgR− phenotype at a rate of 10% year^−1^ for 4 years. Patients with an ER+/PgR− phenotype (either initially or after transition) have twice the recurrence rate on tamoxifen (4 *vs* 2% year^−1^).

**Figure 3:**
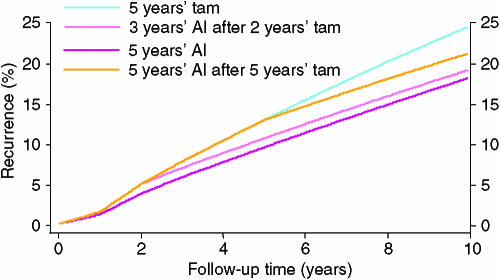
Proportion of oestrogen-receptor-positive women who will develop recurrence in the first 10 years of follow up for four different treatment strategies using the ‘deep model’ and a 5 year carryover effect, assuming 20% of the patients are initially ER+/PgR−: aromatase inhibitor for 5 years; 2 years of tamoxifen followed by 3 years of an AI; 5 years of tamoxifen followed by 5 years of an AI; and 5 years of tamoxifen alone. See text for parameter estimates.

In the ATAC trial, PgR-negative patients had a particularly poor outcome if treated with tamoxifen. This is in keeping with other studies in which PgR negativity is a poor prognostic variable in tamoxifen-treated patients (Bardou *et al*, 2003). In addition, Gross *et al* (1984) have shown that loss of PgR was an early indicator of progression in tamoxifen-treated patients with metastatic disease. This suggests that there is a pressure toward phenotypic shift of micro metastases from PgR+ to PgR− during tamoxifen treatment. If this were true, the differences seen with sequential treatment would not represent ‘tamoxifen priming’, which is difficult to understand, but would reflect a drift towards progesterone-receptor negativity and a more rapid development of resistance with this drug. A simple Markov model using two compartments, based on PgR status and hypothesizing a transition from PgR+ to PgR− in the micro metastases with tamoxifen treatment and a higher recurrence rate in PgR-negative women receiving tamoxifen could explain these data. This is illustrated in Figure 2. In particular, we assume the transition from PgR+ to PgR− is 10% year^−1^ for 4 years and that recurrence rates are the same for AIs or tamoxifen when the phenotype of the micro metastases is ER+/PgR+ (nominally 2% year^−1^), but is twice as high on tamoxifen (4% year^−1^), but unchanged for an AI (2% year^−1^), when the current phenotype is ER+/PgR−. This leads to the results shown in Figure 3 and Table 2 when 20% of the patients are PgR-negative at the outset. A good fit to the observed data and surface model is obtained for the first 6 years, but this model does not predict a long-term benefit for the switching strategy.

In particular, this model suggests that it is better to use an AI first since the effect of using tamoxifen is not to prime patients, but to shift a proportion of them to a phenotype which will progress more rapidly if left on tamoxifen.

## CONCLUSIONS

It must be remembered that these predictions are model-based and will require empirical confirmation. The difference in averaged hazard ratios between the upfront and switching strategies is not statistically significant when a heterogeneity test is employed. In addition to the uncertainties in the estimates of the individual hazard ratios, a more important source of uncertainty is the accuracy of the structural model for extrapolation beyond currently available follow-up times, and the lack of direct comparisons of an AI upfront *vs* a sequencing strategy. The deep model nicely explains the findings of the ATAC and ABCSG8/ARNO95 trials (ATAC Trialists' Group, 2005; Jakesz *et al*, 2005a), where the relative benefit of anastrozole compared to tamoxifen is the same for PgR-negative patients, regardless of when it is started. It also predicts a greater apparent benefit for anastrozole after 2 years of tamoxifen in PgR-positive women because in this period some of these women would have been shifted into a PgR-negative phenotype where tamoxifen would have been particularly less effective. Again, this accords well with the observed data in these trials, and emphasizes the need to consider the whole follow-up period, not just the follow-up after switching, as has been done in the current sequencing trials. The greater benefit of an AI in ER+/PgR− patients compared to ER+/PgR+ tumours was significant in the ATAC trial (Dowsett *et al*, 2005), and was supported by the ABCSG/ARNO trial (Jakesz *et al*, 2005a) and to some extent by the IES trial (Coombes *et al*, 2004b). However, the relatively small number of patients in the ER+/PgR− subgroup and the apparent lack of difference of effect according to PgR status in BIG 1–98 indicate that these predictions need further empirical validation. It is possible that the lack of heterogeneity in the BIG 1–98 trial reflects a difference in the AIs, but the most likely explanation for this phenomenon is a differential effect of tamoxifen in these two subgroups. Bardou *et al* (2003) have found that PgR negativity is an indicator of poor prognosis in tamoxifen-treated women, and this is in accord with several reports that HER-2-positive patients (which are more likely to be PgR-negative) do less well on tamoxifen. However, the EBCTCG overview (Early Breast Cancer Trialists' Collaborative Group 2005) results do not indicate this differential effect in tamoxifen-treated women. This could represent dilution due to heterogeneous and less accurate receptor measurements in the overview, but again this requires further explanation.

If PgR is predictive, then initial use of an AI is clearly indicated for PgR-negative tumours. For PgR-positive tumours, there is more uncertainty, but initial use of an AI, for say 3 years, may still be better than sequencing it after 2 years of tamoxifen, as this is the period with highest recurrence rates. It also avoids the side effects associated with tamoxifen. Trials comparing these two approaches would be highly desirable.

Direct evidence for a phenotype shift, especially in tamoxifen-treated patients may be obtainable by comparing recurrent disease with the primary cancer or monitoring PgR status in peripheral blood as has been done for HER-2 (Meng *et al*, 2004).

Recently Punglia *et al* (2005) have published a model of similar structure to our surface model, but it has important differences in detail, which led them to the conclusion that a switching strategy is optimal. A major difference is that they have used a mixture of end points – DFS for the ATAC, IES BIG 1–98 and ITA trials, but time to recurrence for MA-17 and ABCSG8/ARNO95. These end points are not interchangeable and because of the dilutionary effect of deaths without recurrence when using DFS, this approach will exaggerate the benefits of a switching strategy. Even DFS is defined differently in the trials where they have used it (BIG 1–98 and ITA include any (nonbreast) cancer, but the others do not). For these reasons and the ones stated in the Methods section, time-to-recurrence is the most appropriate end point for these analyses. Punglia *et al* (2005) also explore ‘carryover’ effects in which AIs are assumed to do worse than tamoxifen after both treatments have stopped. This unlikely scenario also leads to a more unfavourable outcome for the initial use of AIs.

Our assumptions on carryover do not have a major effect for the first 10 years of follow-up, except for the extended treatment strategies, which would appear more favourable if there was no carryover. Of course, the duration of treatment is longer here, and a fairer comparison would be against upfront or sequencing strategies with the same duration of treatment. If this were done, the model again predicts that an AI upfront strategy is optimal. Optimal duration of treatment with an AI is a key outstanding question for which there are currently no data. Hopefully this will be addressed in future trials.

Currently, there are also no data for the key comparison of an AI upfront *vs* sequencing after tamoxifen. The BIG 1–98 trial is scheduled to provide such data in 2008 for letrozole and the TEAM study will have data for exemestane, but this is likely to mature even later.

These gaps in our knowledge provide the rationale for the modelling we have done, but again we emphasize that models should be used as interim measures to guide current thinking and practice. They will be superseded by the actual trial data once it becomes available.

Acceptability and side effects are also part of an overall evaluation. Overall, the AIs are better tolerated than tamoxifen, with fewer hot flushes, gynaecologic symptoms, and thromboembolic events, but more joint symptoms and fractures (Coombes *et al*, 2004a; ATAC Trialists' Group, 2005; BIG 1–98 Collaborative Group, 2005). For most women, this would favour using an AI initially, but the differential side-effect profiles may lead to different decisions in individual cases.

## Figures and Tables

**Table 1:** Adjuvant trials of AIS

				**Hazard ratio for recurrence (and 95% CIs where available)**		
**Trial**	**AI**	**Sample size**	**Median follow-up (months)**	**All ER+**	**ER+/PgR+**	**ER+/PgR−**
Initial treatment for up to 5 years						
ATAC^b^^,^^c^	ANA	5216#	68	0.74 (0.64–0.87)	0.84 (0.69–1.02)	0.43 (0.31 –0.61)
BIG 1–98^d^	LET	8028	26	0.72 (0.61–0.86)	0.72^a^	0.72^a^
						
Switching after 2–3 years *vs* continued tamoxifen for 5 years						
IES^e^^,^^f^	EXE	4742	31	0.70 (0.58–0.83)	0.72^a^^*^	0.63**
ITA^g^	ANA	448	52	0.43 (0.25–0.73)	—	—
ABCSG8/ARNO^h^	ANA	3224	28	0.60 (0.44–0.81)	0.66 (0.46–0.93)	0.42 (0.19–0.92)
						
Extended treatment *vs* placebo after 5 years of tamoxifen						
MA-17^i^^,^^i^	LET	5157	29	0.57 (0.43–0.75)	—	—
ABCSG 6a^k^	ANA	856	60	0.64 (0.41–0.99)	—	—

a Based on similar DFS (0.84 *vs* 0.83).

** Based on DFS values of 0.66 *vs* 0.58 in the earlier analysis^e^;

# HR +ve pts.

ANA – anastrozole, LET – letrozole, EXE – exemestane.

b ATAC Trialists' Group, 2005.

c Dowsett *et al*, 2005.

d BIG 1–98 Collaborative Group, 2005.

e Coombes *et al*, 2004a.

f Coombes *et al*, 2004b.

g Boccardo *et al*, 2005.

h Jakesz *et al*, 2005a.

i Goss *et al*, 2003.

j Goss *et al*, 2005.

k Jakesz *et al*, 2005b.

**Table 2:** Recurrence rates at 10 years and ‘time lost to recurrence’ in first 10 years (%) assuming no intercurrent mortality for four different treatment strategies and either a 2 year or 5 year carryover effect

		**Recurrence(%)**				**Time lost (%)**			
	**Carryover (years)**	**Initial AI**	**Tam 2 years + AI 3 years**	**Tam 5 years + AI 5 years**	**Tam 5 years**	**Initial AI**	**Tam 2 years + AI 3 years**	**Tam 5 years + AI 5 years**	**Tam 5 years**
All ER+	2	18.9	19.2	18.9	22.9	9.3	9.9	11	12.1
	5	17.3	17.1	18.9	22.9	9	9.6	11	12.1
									
ER+/PgR−	2	21.9	25.5	25.8	34	9.4	13.2	16.3	18.4
	5	16.3	20.2	25.8	34	8.5	12.3	16.3	18.4
									
ER+/PgR+	2	16	15	15	17.8	7.7	7.3	8.2	9
	5	15.2	13.2	15	17.8	7.6	7.1	8.2	9
									
Deep model	2	20.3	21.3	21.2	24.5	9.6	10.5	11.6	12.5
(20% PgR−)	5	18.1	19.1	21.2	24.5	9.2	10.2	11.6	12.5
